# Complaints about chiropractors, osteopaths, and physiotherapists: a retrospective cohort study of health, performance, and conduct concerns

**DOI:** 10.1186/s12998-018-0180-4

**Published:** 2018-04-12

**Authors:** Anna T. Ryan, Lay San Too, Marie M. Bismark

**Affiliations:** 1 Department of Medical Education, Melbourne Medical School, The University of Melbourne, Parkville, VIC Australia; 2 Centre for Health Policy, Melbourne School of Population and Global Health, The University of Melbourne, Parkville, VIC Australia

**Keywords:** Complaints, Disciplinary action, Chiropractors, Osteopaths, Physiotherapists, Practitioners, Regulation, Risk regulation

## Abstract

**Background:**

Recent media reports have highlighted the risks to patients that may occur when practitioners in the chiropractic, osteopathy and physiotherapy professions provide services in an unethical or unsafe manner. Yet research on complaints about chiropractors, osteopaths, and physiotherapists is limited. Our aim was to understand differences in the frequency and nature of formal complaints about practitioners in these professions in order to inform improvements in professional regulation and education.

**Methods:**

This retrospective cohort study analysed all formal complaints about all registered chiropractors, osteopaths, and physiotherapists in Australia lodged with health regulators between 2011 and 2016. Based on initial assessments by regulators, complaints were classified into 11 complaint issues across three domains: performance, professional conduct, and health. Differences in complaint rate were assessed using incidence rate ratios. A multivariate negative binomial regression model was used to identify predictors of complaints among practitioners in these professions.

**Results:**

Patients and their relatives were the most common source of complaints about chiropractors, osteopaths and physiotherapists. Concerns about professional conduct accounted for more than half of the complaints about practitioners in these three professions. Regulatory outcome of complaints differed by profession. Male practitioners, those who were older than 65 years, and those who practised in metropolitan areas were at higher risk of complaint. The overall rate of complaints was higher for chiropractors than osteopaths and physiotherapists (29 vs. 10 vs. 5 complaints per 1000 practice years respectively, *p* < 0.001). Among chiropractors, 1% of practitioners received more than one complaint – they accounted for 36% of the complaints within their profession.

**Conclusions:**

Our study demonstrates differences in the frequency of complaints by source, issue and outcome across the chiropractic, osteopathic and physiotherapy professions. Independent of profession, male sex and older age were significant risk factors for complaint in these professions. Chiropractors were at higher risk of being the subject of a complaint to their practitioner board compared with osteopaths and physiotherapists. These findings may assist regulatory boards, professional associations and universities in developing programs that avert patient dissatisfaction and harm and reduce the burden of complaints on practitioners.

## Background

Amongst the 15 regulated health professions in Australia, three professions deal with musculoskeletal complaints as a major component of their work. Recent media reports have highlighted the risks to patients that may occur when practitioners in the chiropractic, osteopathy and physiotherapy professions provide services in an unethical or unsafe manner [1–3]. Complaints to professional regulators offer one important window into sources of patient harm and dissatisfaction and have the potential to provide guidance for regulatory and educational interventions.

Previous studies have identified differences in the rate of patient complaints between chiropractors, osteopaths, and physiotherapists [4] and between these professions and general practitioners [5, 6]. However, much of this research has been hampered by lack of standardised complaint processes between regulators of different professions. In Australia, all three professions are registered under the umbrella of a national scheme, providing a valuable opportunity to understand the source, nature, and outcomes of complaints about practitioners in these professions, and to identify differences in complaint risk between the professions.

In the current study, we used comprehensive national data on all registered chiropractors, osteopaths, and physiotherapists in Australia, and formal notifications of concern (“complaints”) to regulators about these practitioners, to better understand complaint risk across these three professions. We aimed to compare the risk of complaint among chiropractors, osteopaths, and physiotherapists and to locate any increased risks in specific aspects of clinical practice.

## Methods

### Context

In Australia, chiropractors, osteopaths and physiotherapists are registered by three regulatory boards - the Chiropractic Board, the Osteopathy Board, and the Physiotherapy Board – under the umbrella of a national health practitioner regulation scheme. Together these professions make-up around 6 % of the registered health workforce in Australia. They are the only practitioners (other than medical doctors) permitted to perform manipulation of the cervical spine under the National Law [7]. They are also all recognised under government schemes (with rebates through the Medicare Enhanced Primary Care program, Veterans’ Affairs, Traffic Accident Commission and WorkCover) and by private health insurers.

There are some important contextual differences between the professions. Physiotherapy is the largest and the longest-established of the three professions in Australia [8]. Formal training in physiotherapy began in Australia in 1907, and was initially affiliated with university medical schools and teaching hospitals [9]. While physiotherapists in some states were registered under the masseurs’ registration board, a separate registration board was first established in Western Australian in the 1950’s [9], followed by other states. The requirement for medical referral for physiotherapy treatment was removed in 1976 [9]. Physiotherapy training in Australia covers a broad scope of practice including aged care, musculoskeletal, neurological, post-operative and sports related populations [8, 10]. A substantial proportion of physiotherapists work in the public domain - around 45% according to one survey of graduates [8].

In contrast, chiropractic and osteopathy are smaller professions in Australia and their assimilation into regulatory and university frameworks occurred later [11, 12]. Statutory regulation of chiropractors and osteopaths was established in all states by the mid-1980’s [13], and assimilation into university-based education occurred in the late 1980’s and 1990’s [13, 14]. Patients have never required medical referral to access chiropractic or osteopathic treatment in this country. Spinal pain syndromes are the most common reason for people to consult with osteopathic practitioners [14]. Chiropractic treatment is most often sought as treatment for musculoskeletal conditions [15], however there is a wide spectrum of chiropractic practice ranging from more ideology-based approaches with a focus on spinal manipulation for general health, through to an evidence-based focus on treatment of musculoskeletal conditions [16].

### Data

We obtained and linked two datasets: 1) a register dataset that covered all chiropractors, osteopaths, and physiotherapists registered to practice in Australia between 1 January 2011 and 31 December 2016; and 2) a complaints dataset that covered all health, performance, and conduct concerns about this group of practitioners lodged with health regulators during the same period.

The register dataset was provided by the Australian Health Practitioner Regulation Agency (AHPRA), a national agency working in partnership with national health practitioner boards who have responsibility for overseeing practitioners in 15 health professions. APHRA maintains a register of practitioners. We obtained a de-identified extract containing information on all chiropractors, osteopaths, and physiotherapists registered for any period of time during the study period in all of Australia’s states and territories. The extract included information on the practitioner’s age (provided in 5 year bands e.g., 1970 to 1974), sex, specialty, and state or territory of practice; the geographic remoteness of their practice location (“practice location”); and dates during which they were registered. Practitioners who held registration in more than one profession (163 practitioners) were randomly allocated into one of those professions. We excluded practitioners registered to addresses outside Australia and those who held non-practising registration. In Australia, practitioners are considered to be practicing when using the knowledge and skills of their profession in their employment, so practice includes teaching and academic roles.

Although practitioners from all three professions are registered with the relevant national board, complaints about practitioners in New South Wales (NSW) are managed by the state based Health Professionals Council Authority (HPCA) in conjunction with the Health Care Complaints Commission and, since July 2014, complaints about practitioners in Queensland are made through the Office of the Health Ombudsman (OHO) [17]. The complaints dataset included information on complaints lodged with AHPRA, the HPCA in NSW and the OHO in Queensland during the study period. This dataset included information collected at the time the complaint was lodged (e.g., lodgement date, source of complaint, primary matter raised), as well as information relating to the ensuing adjudication by the relevant national board (e.g. closure date, case outcome). We linked the register data with the complaints data using anonymised, unique identification variables provided by AHPRA and HPCA.

### Measures

The primary matter of concern raised in each complaint was coded into 11 complaint issues and three domains (health, performance, and conduct). Performance-based complaints relate to knowledge, skill, judgement or care that is of a lower standard than can be reasonably be expected. Conduct-based complaints relate to unethical or illegal conduct. Health complaints relate to physical or mental conditions that may impair a practitioner’s ability to practice safely. The coding of complaint issues was based on initial assessments by AHPRA and HPCA staff of the primary issue raised by the complaint: two researchers working independently grouped 149 codes used by these agencies into the 11 complaint issues most commonly raised in relation to these three professions. Any discrepancies were resolved by consensus. These codes were mutually exclusive, with each complaint assigned to only one complaint issue and one domain (listed in Table 3). “Other performance” and “other conduct” categories included issues that occurred relatively infrequently across all three professions, such as infection control or use of medicines.

### Exposure time

Chiropractors, physiotherapists and osteopaths are “exposed” to risks of complaint primarily when they are engaged in clinical practice. If clinical practice time differs substantially across practitioners in different age groups or professions, it may confound any measures of rate ratio. To address this potential confounder, we created a measure of exposure time and adjusted for it in the analyses. We refer to this as practice years.

Specifically, the exposure time measure was estimated at the practitioner level and was a multiplicative function of two variables: (1) the amount of time in the study period each practitioner was registered (denoted as fractions of years); and (2) the average number of clinical hours worked per week (denoted as a fraction of 40 h, including values > 1). The amount of time each practitioner was registered was calculated directly from the AHPRA register data. Our estimate of clinical hours per week was based on publicly reported information from the 2015 Health Workforce Survey, [18] using the average number of hours worked by practitioners of the same profession, sex, and age (Appendix).

### Data analyses

We used counts and percentages to describe the characteristics of practitioners and complaints about them, stratified by profession (chiropractor, osteopath, and physiotherapist). We also used counts and percentages to describe the source, complaint issue, and outcome of complaints. Overall significant difference among profession for each characteristic was analysed using the chi-square test.

As the absolute numbers of complaints regarding osteopaths were low, a robust between-profession comparison involving complaints about osteopaths was not possible. We therefore conducted analyses comparing the incidence rate of complaints about chiropractors and physiotherapists within each of the three domains (health, performance, conduct) and 11 complaint issues (e.g. treatment, communication, procedures). We used incidence rate ratios (IRRs) to indicate the ratio of the complaint rate amongst chiropractors to the complaint rate amongst physiotherapists, after adjusting for practice years.

Finally, we performed multivariate negative binomial regression analysis to assess the associations between profession, age, sex, practice location and complaint risk across the three professions. The regulators in our study routinely collect these variables and they have previously been shown (in research about medical doctors) to be associated with complaint risk [19, 20]. This analysis was also adjusted for practice years and state/territory of practice location. All analyses were conducted using Stata/SE 14.2. The University of Melbourne’s Human Ethics Sub-Committee approved the project (Approval Ethics ID: 1543670.2).

While advertising complaints are primarily dealt with as a statutory offence under the National Law managed by AHPRA, some are referred on to Boards to be dealt with as conduct concerns [21]. Given recent publicity around increased advertising complaints against chiropractors [22], we conducted a sensitivity analysis in which we repeated our analysis (Tables 1, 2, 3, 4 and 5) with advertising complaints excluded. As there were no major differences in our findings we retained the original analyses.

**Table 1:** Characteristics of chiropractors, osteopaths, and physiotherapists

	Workforce		
	Chiropractor (*n* = 5450)	Osteopath (*n* = 2241)	Physiotherapist (*n* = 31,534)
	%	%	%
Age			
< 36	40.4	51.5	52.8
36-45	27.3	26.9	20.2
46-55	16.5	10.3	13.8
56-65	10.0	7.8	9.7
> 65	5.8	3.5	3.5
Sex			
Female	38.4	54.9	68.2
Male	61.6	45.1	31.8
Practice location			
Metropolitan	76.0	80.5	81.5
Regional and remote	24.0	19.5	18.5

**Table 2:** Characteristics of complaints - source

	Chiropractor (*n* = 543)	Osteopath (*n* = 76)	Physiotherapist (*n* = 520)
	%	%	%
Source			
Patient or relative ^a^	47.5	60.5	59.8
Fellow practitioner	16.9	11.8	10.4
Employer	2.0	5.3	7.9
Other (e.g. government department, insurance company, Medicare, police etc.)	33.5	22.4	21.9

^a^Including complaints received via complaints commissions

**Table 3:** Characteristics of complaints - issue

	Chiropractor (*n* = 543)	Osteopath (*n* = 76)	Physiotherapist (*n *= 520)
	%	%	%
Complaint issue			
Performance Issues	29.1	32.9	41.4
Procedures	3.9	2.6	3.7
Treatment	19.9	19.7	21.7
Communication	1.1	0.0	2.1
Assessment / diagnosis	1.3	1.3	5.6
Other performance	3.0	9.2	8.3
Professional conduct issues	68.1	67.1	52.5
Advertising / titles	8.8	6.6	3.3
Sexual boundaries	10.3	17.1	7.9
Fees / honesty	10.7	6.6	8.3
Interpersonal behaviour	9.4	10.5	8.7
Records / reports	4.8	7.9	6.7
Other conduct issues	24.1	18.4	17.7
Health Issues	2.8	0.0	6.2

**Table 4:** Characteristics of complaints - regulatory outcome^a^

	Chiropractor (*n* = 543)	Osteopath (*n* = 76)	Physiotherapist (*n* = 520)
	%	%	%
Regulatory Outcome ^b c^			
No regulatory action	61.6	56.8	70.0
Referral to another body	1.9	13.5	3.9
Caution, reprimand, fine, or undertaking	15.1	8.1	13.4
Conditions, suspension, or cancellation	21.3	21.6	12.7
Median time to resolution (interquartile range)	213 days (84-422 days)	123 days (67-410 days)	129 days (65-309 days)

^a^Regulatory outcomes not included for NSW data

^b^Does not sum to 100% as the outcome was unknown for a small number of complaints

^c^Excludes cases that were still open at the end of the study period (29% for chiropractors, 11.9% for osteopaths, 11.8% for physiotherapists)

**Table 5:** Predictors of complaints about chiropractors, osteopaths, and physiotherapists

	All complaints	*p*-value^a^
Profession		< 0.001
Chiropractor	4.45 (3.83 – 5.16)	
Osteopath	1.82 (1.38 – 2.40)	
Physiotherapist	1.00	
Age Group		< 0.001
≤ 35	1.00	
36-45	1.63 (1.37 – 1.94)	
46-55	1.76 (1.46 – 2.14)	
56-65	1.92 (1.54 – 2.40)	
≥66	2.28 (1.62 – 3.21)	
Sex		< 0.001
Female	1.00	
Male	2.43 (2.10 – 2.82)	
Practice location		0.031
Metropolitan	1.23 (1.02-1.48)	
Regional and remote	1.00	

^a^This model was adjusted for practice years and state/territory of practice location, and *p*-values represent overall significance difference for each predictor after the adjustment

## Results

### Characteristics of manual therapists and their complaints

During the study period there were 5450 chiropractors, 2241 osteopaths, and 31,534 physiotherapists registered to practice in Australia (Table 1). Practitioners in these three professions differed with respect to age, sex and practice location (*p* < 0.001). Chiropractors were more likely to be aged over 36 years (chiropractors: 59.6%, osteopaths: 48.5% and physiotherapists 47.2%), male (chiropractors: 61.6%, osteopaths: 45.1% and physiotherapists 31.8%), and to practice in non-urban areas (chiropractors: 24.0%, osteopaths: 19.5% and physiotherapists 18.5%).

Across all three professions, more than 90% of practitioners were not subject to any complaints to regulators during the study period (92.3% of chiropractors, 97.1% of osteopaths and 98.5% of physiotherapists had no complaints to regulators.) A total of 1139 complaints about chiropractors, osteopaths and physiotherapists were lodged during the study period.

As a group, chiropractors had a higher overall rate of complaints (29 complaints per 1000 practice years) than osteopaths and physiotherapists (10 and 5 complaints per 1000 practice years respectively, *p* < 0.001). Overall, nearly half of the complaints (47.7%) involved chiropractors, even though chiropractors make up less than one-sixth (13.9%) of the workforce across these three professions.

A small group of practitioners were the subject of multiple complaints. Just over 1 % of chiropractors (1.3%, *n* = 70) were subject to more than one complaint: these practitioners were responsible for over a third (35.5%) of the complaints about chiropractors. Among osteopaths, 0.4% (*n* = 9) accounted for 26.3% of complaints, and among physiotherapists 0.1% (*n* = 40) accounted for 18.7% of complaints.

### Characteristics of complaints

The proportion of complaints lodged by patients or their relatives, fellow practitioners, and employers differed by profession (Table 2). Across all three professions, the most common source of complaints was patients or their relatives (chiropractors: 47.5%, osteopaths: 60.5% vs. physiotherapists: 59.8%). However, a larger proportion of complaints about chiropractors came from fellow practitioners (chiropractors: 16.9% vs. physiotherapists: 10.4%) whereas a smaller proportion of complaints about chiropractors were lodged by employers (chiropractors: 2.0% vs. physiotherapists: 7.9%).

Among chiropractors and osteopaths, more than two-thirds of complaints (68.1% and 67.1%) raised concerns about the practitioner’s professional conduct; approximately one third (29.1% and 32.9%) related to performance issues; and few (2.8% among chiropractors and none among osteopaths) raised concerns about a possible health impairment (Table 3). In contrast, around half of the complaints about physiotherapists (52.5%) raised concerns about professional conduct issues, while the other half pertained to performance (41.4%) and health (6.2%) concerns. Across all three professions, around one fifth of complaints raised concerns about the treatment provided (chiropractors: 19.9% vs osteopaths: 19.7% vs physiotherapists: 21.7%).

The outcome of complaints varied by profession (Table 4). Among closed complaints, 13.5% of complaints about osteopaths were referred to another body, such as a health complaints commissioner; this was higher than for chiropractors (1.9%) and physiotherapists (3.9%). A higher proportion of complaints about chiropractors and osteopaths resulted in registration conditions, suspension or cancellation (21.3 and 21.6% respectively) compared with physiotherapists (12.7%). Across all three professions, over half of the complaints resulted in no further action. Overall, complaints about chiropractors took longer to resolve than complaints about osteopaths and physiotherapists (chiropractors: median of 213 days [interquartile range, IQR = 84 − 422 days]; osteopaths: median of 123 days [IQR = 67 − 410 days]; and physiotherapists: median of 129 days [IQR = 65 − 309 days]).

### Complaint rates and rate ratios

The overall complaint rate for practitioners in the chiropractic, osteopathy and physiotherapy professions was 8 complaints per 1000 practice years. The complaint rate for chiropractors was three times higher than for osteopaths and six times higher than for physiotherapists (29 vs. 10 vs. 5 complaints per 1000 practice years, *p* < 0.001).

**Fig. 1:**
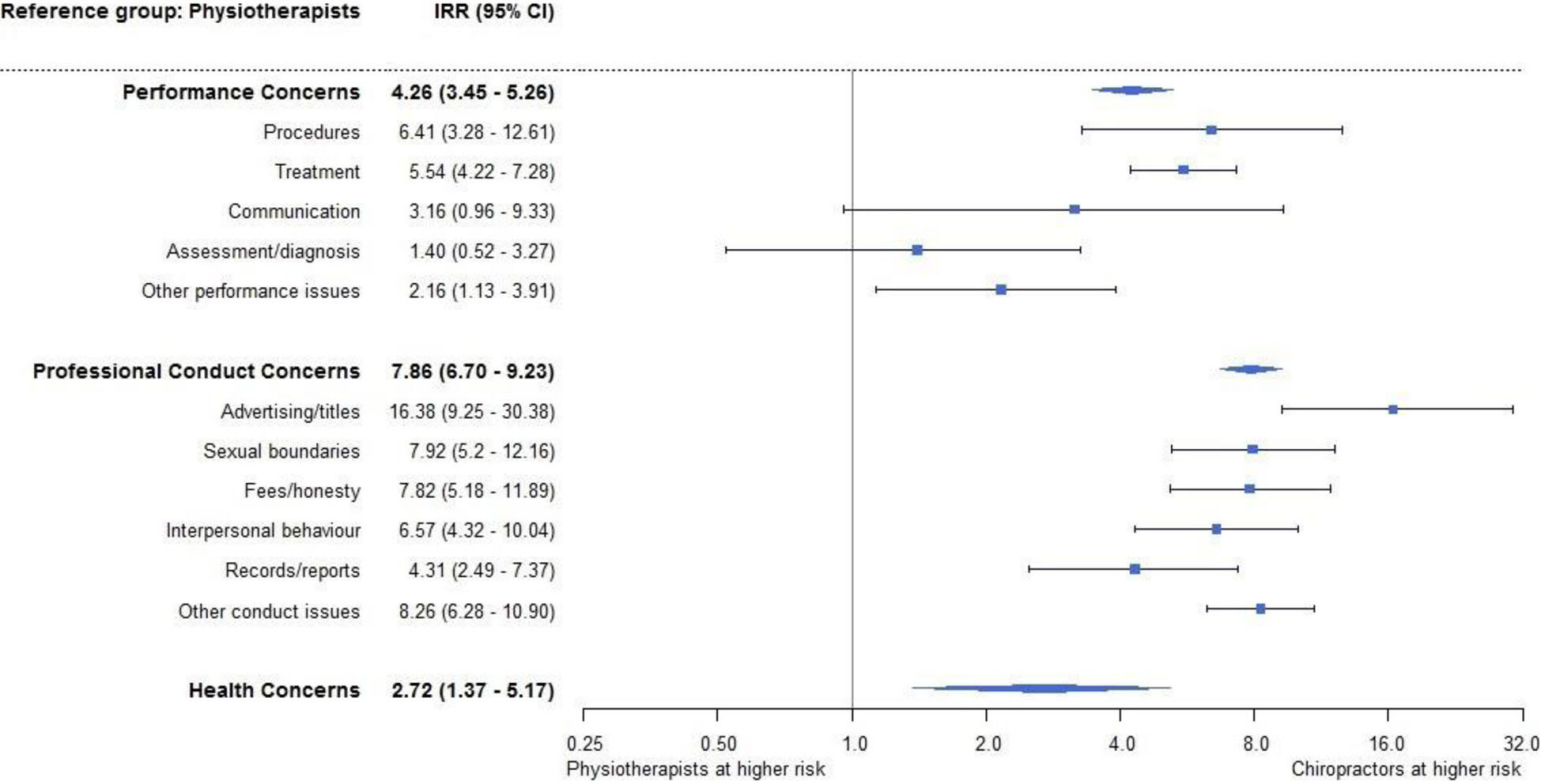
Forest plot of incidence rate ratio of issues comparing chiropractors and physiotherapists

This increased complaint rate spanned all three domains of practice: compared with physiotherapists, the incidence rate of a chiropractor being subject to a complaint was four times higher for performance concerns (IRR = 4.26, 95% CI = 3.45-5.26), around eight times higher for professional conduct concerns (IRR = 7.86, 95% CI = 6.70-9.23), and nearly three times higher for health concerns (IRR = 2.72, 95% CI = 1.37-5.17) (Fig. 1).

Within the 11 specific complaint issues, chiropractors had a higher complaint rate for all classifications, though differences in rates of complaints about assessment/diagnosis and communication were not statistically significant.

The four issues of highest incidence rate for chiropractors, when compared with physiotherapists, were advertising/titles (IRR = 16.38, 95% CI = 9.25-30.38), sexual boundaries (IRR = 7.92, 95% CI = 5.2-12.16), fees/honesty (IRR = 7.82, 95% CI = 5.18-11.89), and interpersonal behaviour (IRR = 6.57, 95% CI = 4.32-10.04). Due to the low absolute number of complaints for osteopaths we did not conduct a similar comparison for osteopaths.

### Predictors of complaints about chiropractors, osteopaths and physiotherapists

Table 5 shows the results of a multivariate analysis of risk factors for complaint across all three professions. Chiropractors had a higher complaint rate compared to physiotherapists (IRR = 4.45, 95% CI = 3.83 – 5.16), after adjusting for age, sex, practice location, practice years as well as state/territory. Across all three professions, practitioners aged 66 years or older had a higher rate of complaints compared to those aged 35 years and younger (IRR = 2.28, 95% CI = 1.62 – 3.21). Male practitioners had 2.4 times the rate of being the subject of a complaint compared with their female peers (IRR = 2.43, 95% CI = 2.10 – 2.82). Practice location was a weaker predictor; those who practiced in metropolitan areas had 1.2 times the rate compared with those who practiced in rural and remote areas (IRR = 1.23, 95% CI = 1.02 – 1.48).

## Discussion

Our aim was to understand differences in the rates and patterns of formal complaints about Australian health practitioners in three health professions: chiropractic, osteopathy and physiotherapy.

During the study period 39,225 practitioners were registered to practice in these professions in Australia. The vast majority of practitioners (greater than 90% in all three professions) were not subject to any complaints to regulators during the study period. Among the 1139 formal complaints about chiropractors, osteopaths and physiotherapists lodged with regulators in Australia over the six-year period, we found that chiropractors had a higher rate of complaints than osteopaths and physiotherapists.

Within the chiropractic profession, over one third of complaints (36%) were about a small number of practitioners (1.3%). This finding that complaints clustered amongst a relatively small number of practitioners is consistent with previous research about medical practitioners [19]. A focused approach to understanding more about this group of practitioners, and assisting them to meet their regulatory obligations could reduce the complaint rate for this profession.

The most common source of complaints for all three professions was patients or their relatives. Nearly 8% of physiotherapy complaints came from their employers, which may reflect the fact that physiotherapists are more likely to work as employees within public sector organisations [8] in comparison to osteopaths and chiropractors. The higher proportion of chiropractic complaints from fellow health practitioners may reflect less inter-professional integration of the profession, anti-competitive behaviour by other practitioners, or the diversity of practice perspectives within the chiropractic professions. There is currently no literature dealing with these factors and so further research is required to explore this.

Within our 11 complaint issues, complaints related to treatment made up around one fifth of all complaints within each profession suggesting this is an important focus of monitoring and education for all three professions. A finer grained classification of concerns about treatment may provide important direction for future research, and subsequent work in education and remediation - for example, a UK study of osteopathic complaints found clinical care concerns were most commonly around inappropriate treatment, or treatment not justified, forceful treatment, treatment that caused new or increased pain or injury, and treatment administered incompetently [23].

Around two-thirds of the complaints about chiropractors and osteopaths raised concerns about professional conduct. One-fifth of complaints about chiropractors related to advertising or fees/honesty. This may reflect increased involvement in private practice – in other words, a heightened level of exposure to such complaints that we could not control for – or inappropriate practice building strategies and small business financial pressures, and this provides an important direction for work in the profession. More than one quarter of complaints about osteopaths related to sexual boundaries or interpersonal behaviour. While absolute numbers are low, measures to monitor and address these areas are likely to be of importance to the osteopathic profession.

We identified differences in regulatory outcomes between the professions: in particular, complaints about osteopaths were more likely to be referred to another agency while complaints about chiropractors took longer to resolve, and were more likely to result in serious outcomes, such as imposition of conditions or suspension of registration, than those involving physiotherapists. Further work is required to understand whether this reflects unintended variation in decision-making between the Boards, or differences in the severity of complaints received about these professions. While over half the complaints across all three professions resulted in no further action, it is important to note that no further action does not necessarily mean a complaint had no substance: it is a common outcome in situations where a practitioner has already taken appropriate steps to address the concerns raised by the complainant.

The higher rate of complaints about chiropractors (compared with physiotherapists), relating to advertising/titles, fees/honesty, sexual boundaries, and interpersonal behaviour (such as bullying and disrespect) could provide useful direction for future research, and highlights the importance of ethics and professionalism in clinical practice.

The finding that older and male practitioners had a higher rate of complaints than female practitioners is consistent with Australian and international studies in medicine [19, 20, 24]. While older practitioners offer a wealth of experience and expertise, the wisdom of age may be eroded by declining health and out-of-date practices. In the field of medicine, several countries have begun to implement measures to assess and support the practice of older doctors [25].

In relation to the difference between male and female practitioners, our analyses controlled for age, duration of registration to practice, and hours of practice, suggesting that the increased complaints rate among male practitioners is not simply due to differences in the practitioner population or time spent in clinical practice. Previous research with medical doctors has identified differences in communication skills between male and female practitioners, which may account for some of these differences [26].

### Study strengths and weaknesses

A key strength of our study is its comprehensiveness. The analysis covered every registered chiropractor, osteopath and physiotherapist in all states and territories in Australia over a six-year study period. Normally, such breadth comes with a substantial trade-off in depth. However, the detailed data on practitioner demographic characteristics and the complaints lodged allowed us to disaggregate complaint rates in a variety of ways, while accounting for clinical hours worked and duration of registration.

Our study has three main limitations. First, complaints are an imperfect indicator of quality of care; they offer only one window into concerns about clinical practice. Previous research suggests that most instances of poor performance, impairment, or unethical conduct do not result in a formal complaint [19]. Second, regulatory staff coded the issues involved in complaints when they were received, based on the information known at the time; this coding does not reflect new information revealed during subsequent assessment and adjudication processes, meaning that issues which are commonly raised as secondary matters – such as communication or record keeping – are likely to be underrepresented in our findings. Other practitioner variables such as patient volume, practice type (including solo or multidisciplinary nature), history of disciplinary action, and country of training may have provided further insights. In particular, we note that a greater proportion of physiotherapists work in the public sector [8] across a range of clinical roles; this may explain at least some of the differences in complaint rates we observed. Finally, due to low numbers within each type of complaint (and given adjusted models did not converge) we used unadjusted models for the incidence rate ratio analysis for chiropractors and physiotherapists (presented in Fig. 1). In addition, due to the very low number of complaints about osteopaths we were unable to do robust comparisons of specific complaint issues at this time; as the national scheme accrues more data such analyses would be worth revisiting.

## Conclusions

Health practitioner regulators are charged with protecting the public from harm [27]. Our aim was to explore the distribution and characteristics of complaints about practitioners in three regulated professions – chiropractic, osteopathy, and physiotherapy - in Australia.

We found differences in the frequency of complaints by issue within the professions. Male practitioners, and those aged 65 years or older had a higher complaint rate independent of their profession. Chiropractors had a higher complaint rate for being the subject of a complaint or concern to their practitioner board compared with osteopaths and physiotherapists. Hotspots of complaint risk for chiropractors (and potential areas for educational and regulatory focus) include advertising/titles, fees/honesty, sexual boundaries and interpersonal behaviour.

Our study highlights a number of areas for future research both within and across the professions. In particular, the impact of profession-specific techniques, patient volume, group practice, interdisciplinary integration, and evidence-based approaches to practice are important foci for future research on complaint risk in these professions.

Programs designed to address hotspots of complaint risk for these professions may help to avert patient dissatisfaction and harm and reduce the burden of complaints on practitioners. Regulatory boards, professional associations and educational bodies may find these data helpful in designing further research and developing interventions that support practitioners in the chiropractic, osteopathy and physiotherapy professions to improve the experience of their patients.

## Appendix

**Table 6:** Predicted working hours

Profession	Age Group	Sex	Predicted working hours per week
Chiropractor	< 20	Female	26.2
Chiropractor	20 - 34	Female	26.2
Chiropractor	35 - 44	Female	21.6
Chiropractor	45 - 54	Female	24.5
Chiropractor	55 – 64	Female	21.4
Chiropractor	65 – 74	Female	22.6
Chiropractor	≥ 75	Female	9.4
Chiropractor	< 20	Male	32.0
Chiropractor	20 - 34	Male	32.0
Chiropractor	35 - 44	Male	32.3
Chiropractor	45 - 54	Male	32.1
Chiropractor	55 – 64	Male	30.0
Chiropractor	65 – 74	Male	24.3
Chiropractor	≥ 75	Male	19.4
Osteopath	< 20	Female	28.5
Osteopath	20 - 34	Female	28.5
Osteopath	35 - 44	Female	22.7
Osteopath	45 - 54	Female	26.4
Osteopath	55 – 64	Female	26.5
Osteopath	65 – 74	Female	19.3
Osteopath	≥ 75	Female	19.3
Osteopath	< 20	Male	33.8
Osteopath	20 - 34	Male	33.8
Osteopath	35 - 44	Male	36.8
Osteopath	45 - 54	Male	35.8
Osteopath	55 – 64	Male	30.6
Osteopath	65 – 74	Male	28.8
Osteopath	≥ 75	Male	21.0
Physiotherapist	< 20	Female	33.6
Physiotherapist	20 - 34	Female	33.6
Physiotherapist	35 - 44	Female	23.1
Physiotherapist	45 - 54	Female	24.4
Physiotherapist	55 – 64	Female	25.2
Physiotherapist	65 – 74	Female	18.6
Physiotherapist	≥ 75	Female	18.5
Physiotherapist	< 20	Male	36.8
Physiotherapist	20 - 34	Male	36.8
Physiotherapist	35 - 44	Male	35.2
Physiotherapist	45 - 54	Male	35.6
Physiotherapist	55 – 64	Male	34.6
Physiotherapist	65 – 74	Male	25.5
Physiotherapist	≥ 75	Male	24.0

## Abbreviations

AHPRA: Australian Health Practitioner Regulation Agency
HPCA: Health Professionals Council Authority
IQR: Interquartile range
NSW: New South Wales
OHO: Office of the Health Ombudsman
